# Dynamic Responses of Female Volunteers in Rear Impact Sled Tests at Two Head Restraint Distances

**DOI:** 10.3389/fbioe.2021.684003

**Published:** 2021-06-08

**Authors:** Anna Carlsson, Stefan Horion, Johan Davidsson, Sylvia Schick, Astrid Linder, Wolfram Hell, Mats Y. Svensson

**Affiliations:** ^1^Chalmers Industrial Technology (Chalmers Industriteknik), Gothenburg, Sweden; ^2^Institute for Legal Medicine, Ludwig-Maximilians-Universitaet (LMU), Munich, Germany; ^3^Vehicle Safety Division, Chalmers University of Technology, Gothenburg, Sweden; ^4^Swedish National Road and Transport Research Institute (VTI), Gothenburg, Sweden

**Keywords:** crash testing, females, soft tissue neck injury, rear impact, sled testing, vehicle safety, volunteers, whiplash

## Abstract

The objective of this study was to assess the biomechanical and kinematic responses of female volunteers with two different head restraint (HR) configurations when exposed to a low-speed rear loading environment. A series of rear impact sled tests comprising eight belted, near 50th percentile female volunteers, seated on a simplified laboratory seat, was performed with a mean sled acceleration of 2.1 g and a velocity change of 6.8 km/h. Each volunteer underwent two tests; the first test configuration, HR10, was performed at the initial HR distance ∼10 cm and the second test configuration, HR15, was performed at ∼15 cm. Time histories, peak values and their timing were derived from accelerometer data and video analysis, and response corridors were also generated. The results were separated into three different categories, HR10_*C*_ (*N* = 8), HR15_*C*_ (*N* = 6), and HR15_*N*__*C*_ (*N* = 2), based on: (1) the targeted initial HR distance [10 cm or 15 cm] and (2) whether the volunteers’ head had made contact with the HR [Contact (C) or No Contact (NC)] during the test event. The results in the three categories deviated significantly. The greatest differences were found for the average peak head angular displacements, ranging from 10° to 64°. Furthermore, the average neck injury criteria (NIC) value was 22% lower in HR10_*C*_ (3.9 m^2^/s^2^), and 49% greater in HR15_*N*__*C*_ (7.4 m^2^/s^2^) in comparison to HR15_*C*_ (5.0 m^2^/s^2^). This study supplies new data suitable for validation of mechanical or mathematical models of a 50th percentile female. A model of a 50th percentile female remains to be developed and is urgently required to complement the average male models to enhance equality in safety assessments. Hence, it is important that future protection systems are developed and evaluated with female properties taken into consideration too. It is likely that the HR15 test configuration is close to the limit for avoiding HR contact for this specific seat setup. Using both datasets (HR15_*C*_ and HR15_*N*__*C*_), each with its corresponding HR contact condition, will be possible in future dummy or model evaluation.

## Introduction

Today, low-to-moderate speed rear impact testing is performed with 50th percentile male dummies, mainly with the BioRID II, which limits the assessment and development of whiplash protection systems with regard to female occupant protection (Linder and Svensson, 2019). In terms of stature and mass, the 50th percentile male crash test dummy roughly corresponds to the 90th–95th percentile female (Welsh and Lenard, 2001), resulting in females not being well represented by the existing low velocity rear impact male dummy; BioRID II. Accident data shows that females have a greater risk of sustaining whiplash injuries than males, even under similar crash conditions (Kihlberg, 1969; O’Neill et al., 1972; Otremski et al., 1989; Morris and Thomas, 1996; Temming and Zobel, 1998; Chapline et al., 2000; Krafft et al., 2003; Storvik et al., 2009; Carstensen et al., 2011). According to these studies, the whiplash injury risk is up to three times higher for females compared to males.

Passenger vehicles equipped with advanced whiplash protection systems posed on average a ∼50% lower risk of long-term whiplash injuries for occupants in rear impacts, than for occupants in passenger vehicles manufactured after 1997, without whiplash protection systems installed (Kullgren et al., 2007). Nevertheless, insurance data show that whiplash injuries account for 63% of all injuries leading to permanent medical impairment sustained in passenger vehicles on the Swedish market (Gustafsson et al., 2015). In rear impacts, the risk reduction for permanent medical impairment is approximately 30% greater for males than for females according to insurance claims records (Kullgren and Krafft, 2010), which effectively means that the difference between female and male whiplash injury risk has increased, although the general whiplash injury risk has reduced. In recent years, injury statistics show that whiplash injuries still present a major problem, and that the whiplash injury risk females are exposed to is substantially higher (Kullgren et al., 2020).

Low-speed rear impact volunteer tests have shown that females have greater horizontal head accelerations, greater (or similar) horizontal T1 accelerations, lesser head and T1 rearward displacements, lesser (or similar) Neck Injury Criterion (NIC) values, and more pronounced rebound motions in comparison to males (Siegmund et al., 1997; Mordaka and Gentle, 2003; Viano, 2003; Ono et al., 2006; Linder et al., 2008; Schick et al., 2008; Carlsson et al., 2011, 2012). The results show that there are characteristic differences in the dynamic response between males and females in rear impacts.

Based on mathematical simulations, Mordaka and Gentle (2003) concluded that a “scaled down male model is not adequate to simulate female responses even though the scaling constitutes a good height and mass match” (p. 52). Additionally, Vasavada et al. (2008) found that “male and female necks are not geometrically similar and indicate that a female-specific model will be necessary to study gender differences in neck-related disorders” (p. 114). That is, a female model must be based on data from tests with females.

The greatest whiplash injury frequencies are associated with females and males of average statures (Kihlberg, 1969; Carlsson et al., 2014). Based on US injury statistics, the highest whiplash injury frequency was recorded for the statures 162.6–165.1 cm (64–65 in) for the females and 175.3–177.8 cm (69–70 in) for the males, both close to the average statures of the US population (females: 161.8 cm (63.7 in), males: 175.3 cm (69.0 in); Schneider et al., 1983). Based on Swiss and Swedish insurance records, Carlsson et al. (2014) concluded that the stature and mass of the females most frequently injured correspond well with the average stature and mass of the female populations in these countries.

Hence, there is a need for 50th percentile female models, physical and/or computational crash test dummies and human body models (HBMs), to further improve the vehicle safety for both females and males (Carlsson, 2012; Carlsson et al., 2017). Human dynamic response data is important when developing and evaluating such occupant models. Thus, the objective of this study was to generate dynamic response data and investigate differences in seat interaction for near 50th percentile females in a laboratory seat at two different head restraint (HR) configurations.

## Materials and Methods

A series of rear impact sled tests comprising female volunteers was performed at a velocity change of ∼7 km/h with two nominal HR distances. The test series was approved by the ethical committee at the Ludwig-Maximilian University in Munich, Germany, Approval Reference Number 319-07 (Address: Ethikkommission der Medizinischen Fakultät der LMU, Pettenkoferstr. 8a, 80336 Munich, Germany).

### Volunteers

Female volunteers were recruited by advertisements at the Ludwig-Maximilian University in Munich, Germany. Potential subjects were preselected by telephone interviews. Exclusion criteria included any known histories of spinal symptoms; former whiplash associated disorders (WADs); former fractures and/or surgical interventions to the vertebral column; familial or hereditary spinal disorders, disc protrusion or herniations, rheumatism and rheumatoid diseases, further orthopaedic diseases, syndromes and symptoms such as, arthrosis, arthritis, multiple cartilaginous exostoses, scoliosis, spondylolisthesis; having been under treatment (massage/non-steroidal anti-inflammatory drugs/exercises/chiropractic or other therapies) for the back/neck during the 6 months preceding the tests. The volunteers were examined by a physician prior to the tests and further exclusions were made based on these objective findings or if subjective discomfort in the head/neck/shoulders/back existed on the test day. Anthropometric data were obtained on the same occasion.

Eight female volunteers participated in the test series. Their age ranged between 22 and 29 years at an average of 26 years, their stature ranged between 161 and 166 cm at an average of 163 cm, and their mass ranged between 55 and 67 kg at an average of 60 kg (Table 1). According to the University of Michigan Transportation Research Institute (UMTRI), the stature and mass of the 50th percentile female is 162 cm and 62 kg, respectively, (Schneider et al., 1983). In comparison to the UMTRI data, the female volunteers were on average 1% taller and 4% lighter than the 50th percentile female.

**TABLE 1:** The age, stature, mass, Δv and head-to-HR distance of the individual female volunteers (A–H), as well as their average values and standard deviations (SD).

**Test subject**				**HR10**		**HR15**	
				**Initial HR**		**Initial HR**	
				**distance 10 cm**		**distance 15 cm**	
	**Age [years]**	**Stature [cm]**	**Mass [kg]**	**Δv^*b*^ [km/h]**	**HR distance^*d*^ [cm]**	**Δv^*b*^ [km/h]**	**HR distance^*d*^ [cm]**
A^*a*^	27	161.0	54.5	6.95	12.0	6.89	16.3
B	26	163.8	56.8	6.61	7.8	6.75	14.4
C	27	162.8	66.8	6.73	11.5	6.86	15.3
D	23	166.0	56.8	6.72	9.1	6.87	13.5
E	25	165.3	61.2	6.94	9.2	6.69	14.1
F	29	161.4	62.2	6.89	7.3	6.85	14.2
G	22	161.9	60.4	6.73	7.6	6.88	14.9
H^*a*^	27	164.4	58.0	6.87	11.4	6.87	16.5

Average	**26**	**163.3**	**59.6**	**6.81**	**9.5**	**6.83**	**14.4**
SD^*c*^	**2**	**1.8**	**3.9**	**0.12**	**1.9**	**0.07**	**1.1**

*^*a*^No HR contact at HR15.*

*^*b*^Change of velocity.*

*^*c*^Standard Deviation (SD).*

*^*d*^At impact.*

### Sled and Seat System

**FIGURE 1:**
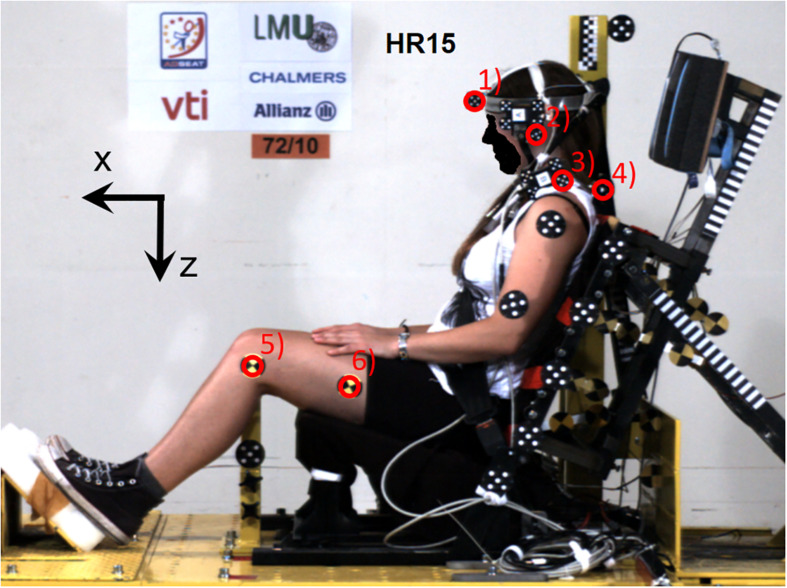
Volunteer test setup; in this case for the head-to-HR distance 15 cm (HR15). Video tracking targets **(1)** and **(2)** for determining head displacements and targets **(3)** and **(4)** for T1 displacements. The position of the trochanter major was palpated and measured prior to the test, and its linear displacement was obtained from targets **(5)** and **(6)**. The thickness of the dark head restraint padding was adjusted to adapt the head-to-head restraint distance for each volunteer, to either 10 cm or 15 cm.

A stationary target sled (1,005 kg) equipped with a laboratory seat was impacted from the rear by a bullet sled (570 kg). The ram-shaped front structure of the bullet sled activated an iron band, mounted inside a band-brake on the target sled, dimensioned to create a predefined acceleration and velocity change of the target sled. The laboratory seat had the same seatback construction as in previous tests series (Davidsson et al., 1998; Carlsson et al., 2011). The seatback was designed to resemble the shape and deflection properties of a Volvo 850 car seat and consisted of four stiff panels covered with 20 mm medium quality Tempur foam. The panels were independently mounted to a rigid seatback frame by coil springs to allow easy implementation into a computational model. The seatback was adjusted to 24.1°. The seat specifications can be found in the Davidsson et al. (1999) publication. In the present study, the HR was modified and consisted of a plywood panel (dimensions: 350 × 230 × 20 mm) covered by firm padding (polyethylene 220-E) and supported by a rigid steel frame, i.e., it was not coupled to the deflecting parts of the seatback. This HR design was chosen to achieve improved reproducibility, based on experience gained in the earlier test series (Carlsson et al., 2011). The HR angle was 12.4° from the vertical plane. The targeted initial head-to-HR distance was set by adjusting the thickness of the padding on the HR for each individual (Figure 1). The HR surface stiffness was not affected by the change in thickness of the padding, typically from 13 to 8 cm. The seat base was rigid and the flat seat surface (dimensions: 500 × 500 × 20 mm) was angled 16.9° from the horizontal plane. A plate was mounted on the sled to resemble a passenger floor pan surface of a car (Figure 1). The seatback and seat base were covered with double layers of knitted lycra fabric.

### Test Procedures and Test Configurations

The volunteers were seated on the laboratory seat, restrained by a 3-point seatbelt and instructed to obtain a natural seated posture, position their feet on the angled plate, place their hands on their lap, face forward and relax prior to the impact. Then, prior to the test, the head-to-HR distance was checked, and the volunteers were reminded to remain seated in a relaxed manner. There was no countdown, and the volunteers were aware of the impending impact since the bullet sled created sound as well as vibrations that could be sensed. Each volunteer underwent two tests; the first test configuration, labelled HR10, was performed at the targeted initial HR distance ∼10 cm, and the second test configuration labelled HR15, was performed at ∼15 cm. These two HR placements were chosen to provide additional distance, in 5 cm increments, compared to the 5 ± 2 cm in the earlier test series of Carlsson et al. (2011). The chosen head-to-HR distances are greater than what is typically found in recent passenger vehicle seats when seated in neutral, upright position (Park et al., 2018). The volunteers were asked to leave the seat for approximately 10 min between the tests. At this point the volunteers were asked if they wanted to proceed with the second test (HR15). This non-randomised order of tests was chosen to allow the volunteers to experience the smaller head-to-HR distance before continuing. A randomised order of the two tests would likely only have had a marginal effect on the head kinematics results. Siegmund et al. (2003) carried out repeated rear impact volunteer tests to study the influence of habituation on the neck response. Their results suggests that the habituation from the first to the second test had negligible influence of the onset phase of the head motion, although at a much lower rear impact severity. The same sled pulse was applied in all tests, with an average mean target sled acceleration of 2.1 g and a velocity change of 6.8 km/h. The volunteers were wearing their own clothes, a pair of shorts and a vest/T-shirt during the tests.

### Instrumentation and Data Acquisition

The head of each volunteer was equipped with a harness which was fixed tightly to the head (Figure 1). Linear tri-axial accelerometers (MSC 322C/AM-100) were mounted on the left side of the harness and an angular accelerometer (Endevco 7302B) on the right side, approximately at the head centre of gravity. Linear accelerometers (Endevco 7264–200) in x- and z-direction were placed on a holder above the T1 vertebra. The holder was attached to the skin at four points; one above each of the proximal ends of the clavicles, and two bilateral and close to the spinal process of the T1 vertebra. The HR contact was measured by a tape switch (Barger 121 BP). Linear accelerometers (Endevco 2262A–200) recorded bullet sled and target sled accelerations in the x-direction. The start of the impact (*T* = 0) was defined by a tape switch (Barger 101 B) attached to the steel bar on the target sled. Video tracking targets were secured on the volunteers prior to the tests (Figure 1).

Two high-speed digital video cameras (Redlake HG100K, 1,504 × 1,128 pixels, 1,000 f/s) monitored the tests from the left side and perpendicular to the direction of the sled tracks; one providing a close-up view and one providing an overview. Both were placed approximately 6.8 m from the midplane of the volunteers. Sensor data was registered by a Kayser-Trede MiniDau acquisition unit at 10 kHz sampling rate and anti-alias filtered at 4 kHz.

### Data Analysis

The sled, head and T1 accelerations were filtered at CFC60, CFC1000, and CFC60, respectively, as defined by SAE J211. Two different accelerometer coordinate systems were defined; their centres were located at respective accelerometer positions and the two systems moved as the position of the volunteer changed during impact. The coordinate systems were defined according to SAE J211 (orthogonal right-handed), with the positive x-, y-, and z-axis forward, rightward and downward, respectively, at the beginning of the impact.

Videos were digitised in Tema 3.5 software. None of the displacement data was filtered. The linear displacements of the head and T1 were obtained from video tracking targets (2) and (4), respectively (Figure 1). The angular displacement of the head was derived from targets (1) and (2) and the T1 from targets (3) and (4). In addition, the position of the trochanter major was palpated and measured prior to testing, and its linear displacement was obtained from targets (5) and (6). The actual head-to-HR distance at the time *T* = 0 was obtained from video analysis, and this distance deviated somewhat from the targeted distance (Table 1). The displacement data was set to zero at the time of impact (*T* = 0) and was expressed in a sled fixed coordinate system.

Peak values and their timing were derived from the data, and response corridors were generated. A Shapiro-Wilks test for statistical normality was performed on the data set. For each dynamic response parameter, we investigated whether the observed differences in parameter values between HR10_*C*_ and HR15_*C*_ were statistically significant. HR15_*N*__*C*_ was excluded from this analysis since this category only involved two samples. *T*-tests were performed with the statistical significance level of .05 with no corrections for multiple comparisons. Response corridors for the volunteers were defined as the average ± 1 standard deviation (SD). The peak values of the head and T1 x-accelerations, x- and angular displacements as well as their occurrence in time were determined for each volunteer. The HR distance was (1) adjusted (pre-test) to 10 and 15 cm, respectively, and (2) estimated from video analysis at impact (*T* = 0). The HR contact time was documented. The NIC value (Boström et al., 1996, 2000) was calculated from SAE J211/1 (2003) standard CFC60 filtered head and T1 accelerations.

## Results

The results were separated into three different categories, HR10_*C*_, HR15_*C*_, and HR15_*N*__*C*_, based on

(1) the targeted initial HR distance (10 or 15 cm) and (2) whether the volunteers’ head had made contact with the HR during the test event [Contact (C) or No Contact (NC)]:

HR10_*C*_: - 8 tests

- Initial HR distance 10 cm
- HR contact
- Represented by dark grey corridors

HR15_*C*_:

- 6 tests
- Initial HR distance 15 cm
- HR contact
- Represented by light grey corridors

HR15_*N*__*C*_:

- 2 tests
- Initial HR distance 15 cm
- No HR contact
- Represented by solid black lines

At the time *T* = 0, the two volunteers (A and H, Table 1) with no HR contact (HR15_*N*__*C*_) were placed in a separate group since they had somewhat greater actual head-to-HR distance (16.3 and 16.5 cm) in comparison to the six volunteers with HR contact (ranging from 13.5 to 15.3 cm). The greater distance may be the reason why no contact occurred. No symptoms from the neck were reported by the volunteers after the tests.

Response corridors were defined as the average ± 1 SD from the average response for the eight female volunteers, except for two cases where no HR contact had occurred. In Supplementary Appendix 1, in the online supplement, each individual response curve is presented together with the corridors.

**FIGURE 2:**
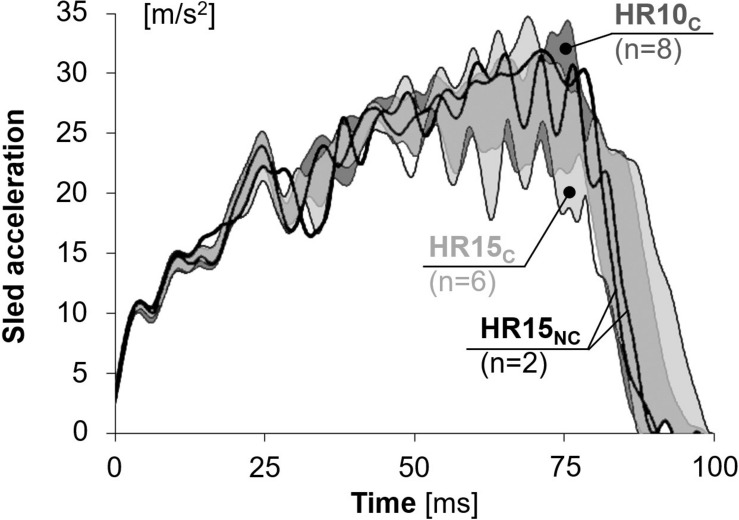
The sled pulse for tests comprising 50th percentile female volunteers.

The average speed change applied was 6.8 ± 0.1 km/h (Figure 2).

### Initial HR Distance and HR Contact

Estimated from video analysis (at *T* = 0), the HR distance was on average 9.5 cm in HR10_*C*_, 14.4 cm in HR15_*C*_, and 16.4 cm in HR15_*N*__*C*_ (Table 2). The HR contact started 23% (*P* = 0.000) and ended 16% (*P* = 0.000) earlier, respectively, in HR10_*C*_ in comparison to HR15_*C*_, however, the length of the HR contact was approximately the same (40 and 37 ms, respectively).

**TABLE 2:** Summary of results from the tests comprising near 50th percentile female volunteers.

	**HR10_*C*_**		**HR15_*C*_**		**HR15_*N*__*C*_**	
	**Initial HR distance 10 cm**		**Initial HR distance 15 cm**		**Initial HR distance 15 cm No**	
	**HR contact (*N* = 8)**		**HR contact (*N* = 6)**		**HR contact (*N* = 2)**	
**Variable**	**Peak**		**Peak**		**Peak**	
	**Average (SD)**	**Time**	**Average (SD)**	**Time**	**Average**	**Time**
**X-Displacement**^*a*^	[mm]	[ms]	[mm]	[ms]	[mm]	[ms]
- Head	–113 (12)	121 (11)	–138 (9)	149 (7)	–133	156
- T1	–96 (9)	127 (5)	–104 (8)	135 (6)	–92	126
- Head relative to T1	–26 (15)	142 (47)	–50 (13)	188 (33)	–100	211
- Trochanter Major	–96 (6)	123 (5)	–94 (3)	122 (5)	–96	124
**Angular displacement**	[°]	[ms]	[°]	[ms]	[°]	[ms]
- Head	10 (9)	140 (44)	28 (9)	202 (13)	64	237
- T1	18 (2)	144 (6)	24 (3)	159 (8)	20	149
- Head relative to T1^*b*^	–12 (6)	131 (18)	–7 (2)	126 (27)	–5	100
- Head relative to T1^*c*^	5 (11)	263 (67)	15 (9)	235 (15)	47	242
**X-Acceleration**	[m/s^2^]	[ms]	[m/s^2^]	[ms]	[m/s^2^]	[ms]
- Head	193 (35)	116 (12)	106 (40)	147 (8)	32	115
- T1	62 (10)	130 (8)	47 (6)	135 (13)	49	132
**NIC**	[m^2^/s^2^]	[ms]	[m^2^/s^2^]	[ms]	[m^2^/s^2^]	[ms]
	3.9 (1.1)	91 (19)	5.0 (2.1)	123 (23)	7.4	134
**Head restraint (HR)**	[mm]	[ms]	[mm]	[ms]	[mm]	[ms]
- Head-to-HR distance^*d*^	95 (19)	–	144 (6)	–	164	–
- Contact (start)	–	99 (12)	–	129 (8)	–	None
- Contact (end)	–	139 (11)	–	166 (7)	–	None

*^*a*^Relative to the sled.*

*^*b*^First peak.*

*^*c*^Second peak.*

*^*d*^At T = 0 ms (based on video analysis).*

### Linear Displacements

**FIGURE 3:**
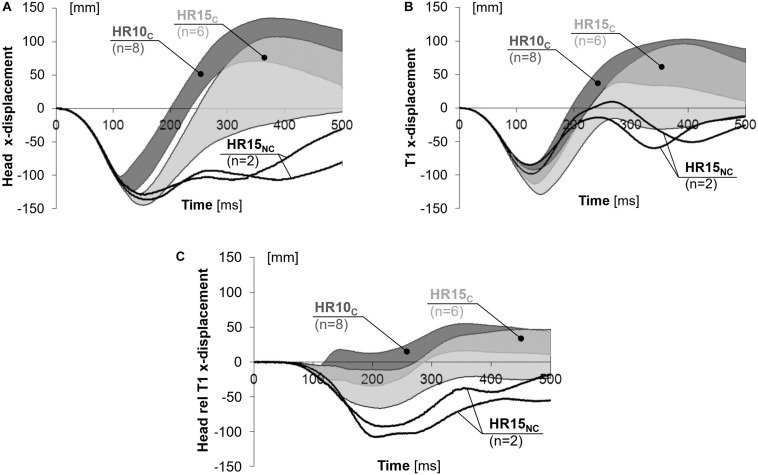
X-displacement of the **(A)** head, **(B)** T1, and **(C)** head relative to T1 for near 50th percentile female volunteers.

**FIGURE 4:**
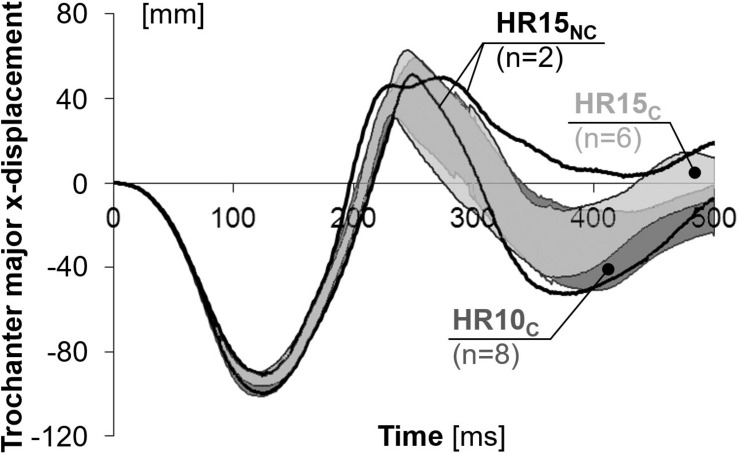
X-displacements of the trochanter major for near 50th percentile female volunteers.

Linear displacements are presented in Figure 3 and Table 2, as well as in Supplementary Figures A1.1–3 in the online supplement. On average, HR10_*C*_ resulted in 18% less (*P* = 0.001) and 19% earlier (*P* = 0.000) peak rearward x-displacement of the head (negative values in Figure 3A) compared to HR15_*C*_. In T1, the peak rearward x-displacement (negative values in Figure 3B) was on average 7% less [not statistically significant (NS)] and 6% earlier (*P* = 0.017) compared to HR15_*C*_. This resulted in substantial differences between the configurations in the rearward x-displacement of the head relative to T1 (negative values in Figure 3C); HR10_*C*_ was on average 48% less (*P* = 0.009) and 24% earlier (NS) in comparison to HR15_*C*_, while HR15_*N*__*C*_ was 102% greater and 12% later, in comparison to HR15_*C*_ (Table 2). In the trochanter major, the rearward x-displacement was similar for the two configurations, HR10 and HR15 (Figure 4 and Table 2).

The rebound motion was most pronounced in HR10_*C*_, with an earlier return to the initial position (= 0 cm) and a greater forward x-displacement after 500 ms (positive values in Figures 3A,B). In HR10_*C*_, the head returned to the initial position on average 39% earlier (*P* = 0.006) in comparison to HR15_*C*_ (217 and 356 ms, respectively). For the two volunteers in HR15_*N*__*C*_ the head did not return to its original position. The entire rebound motion was not captured by the cameras. After 500 ms, the average forward x-displacement of the head was 90% greater (NS) in HR10_*C*_ (76 mm) compared to HR15_*C*_ (40 mm) (Figure 3A). Correspondingly for the T1, the average forward displacement after 500 ms was 61% greater (NS) for HR10_*C*_ (48 mm) than HR15_*C*_ (30 mm), while the T1 lagged behind (–19 mm) for HR15_*N*__*C*_ (Figure 3B).

### Angular Displacements

**FIGURE 5:**
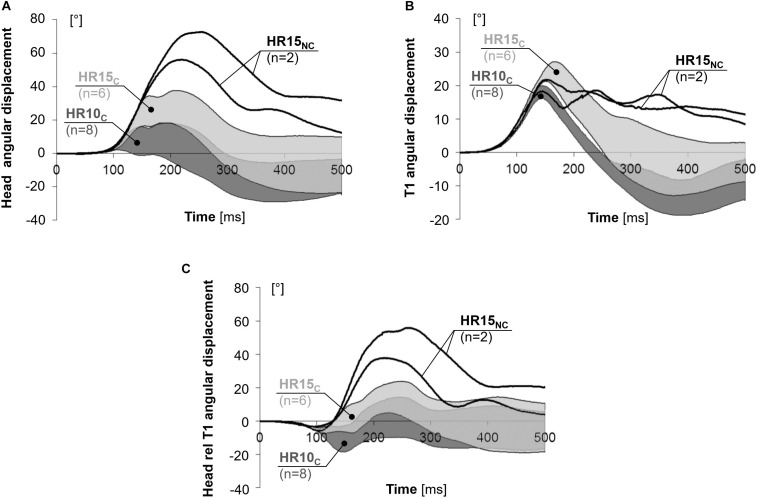
Angular displacement of the **(A)** head, **(B)** T1, and **(C)** head relative to T1 for near 50th percentile female volunteers.

Angular displacements are presented in Figure 5 and Table 2, as well as in Supplementary Figures A1.4–6 in the online supplement. The rearward angular displacements showed substantial differences in the two configurations (positive angles in Figure 5). In comparison to HR15_*C*_, the peak rearward head angular displacement was 64% less (*P* = 0.003) and 31% earlier (*P* = 0.006) in HR10_*C*_ (Figure 5A). For the two volunteers that never made head-to-HR contact in HR15_*N*__*C*_, the peak rearward head angular displacement was 128% greater and 17% later than the other six volunteers in HR15_*C*_. The corresponding numbers for T1 were 25% less (*P* = 0.001) and 9% earlier (*P* = 0.002) in HR10_*C*_ (Figure 5B). Because the rearward angular displacement of T1 started earlier in comparison to the head, the volunteers exhibited a small forward angulation (flexion) of the head relative to T1 during the first ∼100 ms for all HR conditions (negative angles in Figure 5C). In HR10_*C*_, this forward peak head relative to T1 angular displacement was on average 75% greater (NS) in comparison to HR15_*C*_. Furthermore, the early HR contact in HR10_*C*_ resulted in less rearward angulation (extension) of the head relative to T1, whereas in HR15_*C*_, the extension of the head relative to T1 was more prominent. In comparison to HR15_*C*_, the peak rearward head relative to T1 angular displacement was 70% less (NS) in HR10_*C*_ (Table 2).

During the rebound, HR10_*C*_ showed an earlier return of the head and T1 angles to their initial positions (= 0°), and a more pronounced forward flexion after 500 ms (negative angles in Figures 5A,B) compared to HR15. The head returned to the initial position on average 23% earlier in HR10_*C*_ in comparison to HR15_*C*_ (249 and 324 ms, respectively, based on the average curves of the corridors in Figure 5A). In the T1, the corresponding values were 24% earlier in HR10_*C*_ compared to HR15_*C*_ (236 and 312 ms, respectively, based on the average curves of the corridors in Figure 5B). The average curves of the corridors were used due to some of the volunteers not returning to their initial position within the time frame, 500 ms (thus calculating the significance was not meaningful). In HR15_*N*__*C*_, neither the head nor the T1 returned to the initial position in any of the two tests. After 500 ms, HR10_*C*_ showed a 102% larger forward flexion of the head in comparison to HR15_*C*_ (–13.6° and –6.7,° respectively, NS), while in HR15_*N*__*C*_ the head remained in extension (22°). Correspondingly, after 500 ms the T1 angular displacement was on average 179% greater in HR10_*C*_ than in HR15_*C*_ (–8,1° and 2,9°, respectively, NS), while in HR15_*N*__*C*_ the T1 remained in extension (10°).

### Sensor Data

**FIGURE 6:**
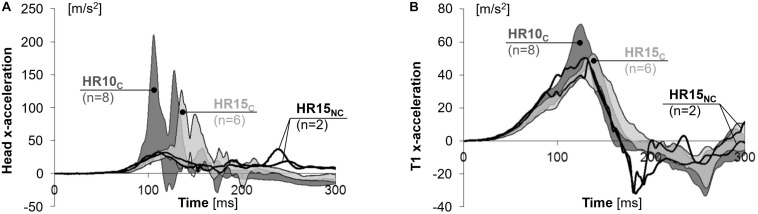
X-accelerations of the **(A)** head and **(B)** T1 for near 50th percentile female volunteers.

Linear head and T1 accelerations are presented in Figure 6 and Table 2, as well as in Supplementary Figures A1.5–12 in the online supplement. The peak head forward x-acceleration was on average 82% greater (*P* = 0.001) and 22% earlier (*P* = 0.000) in HR10_*C*_, and 69% less and 22% earlier in HR15_*N*__*C*_, as compared to HR15_*C*_ (positive values in Figure 6A). In the T1, the peak forward acceleration was on average 34% greater (*P* = 0.004) in HR10_*C*_ compared to HR15_*C*_ (positive values in Figure 6B).

In comparison to HR15_*C*_ (5.0 m^2^/s^2^ at 123 ms), the NIC value was on average 22% lower (NS) and 26% earlier (*P* = 0.015) in HR10_*C*_ (3.9 m^2^/s^2^ at 91 ms), and 49% greater and 9% later in HR15_*N*__*C*_ (7.4 m^2^/s^2^ at 134 ms) (Table 2).

## Discussion

To further improve vehicle safety for both females and males, 50th percentile female models, physical and/or computational crash test dummies and human-body models (HBMs), are required (Carlsson, 2012, 2017). Human dynamic response data is important when developing and evaluating these occupant models. Thus, the objective of this study was to generate response corridors and investigate differences in seat interaction for near 50th percentile females in a laboratory seat at two different HR configurations.

The eight female volunteers participating in the tests were closely matched in size (163.3 ± 1.8 cm/59.6 ± 3.9 kg, Table 1) to the 50th percentile female according to the UMTRI study (162 cm/62 kg, Schneider et al., 1983). It is important to note that the average anthropometry varies between different regions of the world. However, we aimed for an anthropometric definition representative for the world population. The anthropometry study of the WorldSID project (Moss et al., 2000) concluded that the size of a world-harmonised 50th percentile adult male would correspond well with the size of the 50th percentile adult male as defined by the UMTRI project (Robbins, 1983a,b; Schneider et al., 1983). We found it reasonable to make the same assumption regarding the 50th percentile adult female (Carlsson et al., 2014).

The present test setup is based on an earlier setup with an average head-to-HR distance of 5.5 cm for the female volunteers (Davidsson et al., 1999; Carlsson et al., 2011). The new setup was designed to provide a greater initial HR distance in 5 cm increments (10 and 15 cm, respectively). The increased HR distance was introduced to enable larger relative motions between the head and the upper torso. Since the initial HR distance was greater compared to previous test series, the mean sled acceleration was reduced from ∼3 to ∼2 g to ensure the volunteers’ safety. The selection of the reduced mean acceleration was based on a previous study (Krafft et al., 2002), reporting that long-term whiplash injury risks approached 0% for mean vehicle accelerations below 3 g. Krafft et al. presented mean accelerations ranging from 1 to 7 g indicating that the current sled pulse is representative of the lower range of real-world rear impacts. Furthermore, in comparison to previous test series the design of the laboratory seat was simplified to facilitate computational modelling and reproducibility. The earlier Volvo 850 seat base was replaced with a rigid, flat surface. In addition, the earlier spring-mounted HR panel was replaced by a rigid, adjustable construction to obtain a more precise and reproducible position of the HR during impact (Figure 1). The initial HR distance was adjusted by adding padding to the HR, i.e., the geometry was similar for all volunteers in HR10_*C*_ and HR15_*C*_, respectively. Consequently, seatback panels were flexing like a standard car seat, while the HR stayed still relative to the sled during the dynamic event. This seatback and HR design deviates from a typical vehicle front seat, however, in this study, the reproducibility was given priority.

The results from the HR15 test configuration were separated into two groups. In the first group, HR15_*C*_, the head did contact the HR, while in the second group, HR15_*N*__*C*_, HR contact did not occur. It is likely that the HR15 test configuration is close to the limit of avoiding HR contact for this specific seat setup. At the time *T* = 0, the HR15_*N*__*C*_ volunteers had a greater head-to-HR distance (16.3 and 16.5 cm) in comparison to the HR15_*C*_ volunteers (ranging from 13.5 to 15.3 cm). Furthermore, HR15_*N*__*C*_ also had lesser x-displacements of the head (13.3 and 13.8 cm) and T1 (9.2 and 10.4 cm) (Table 2). The greater distance likely explains why no HR contact occurred. The results deviated significantly between the two groups after the time of HR contact in the HR15_*C*_ group (on average 129 ms, Table 2 and Figures 3–6). It will be possible to use both datasets in future dummy and model evaluations, each with its corresponding HR contact condition. The grey corridors of HR15_*C*_ can be used in case the dummy or model contacts the HR (targeting 129 ms), while the black lines of the HR15_*N*__*C*_ can be used in non-contact cases. Together, the two datasets represent parts of the mid-sized female population. When evaluating a dummy or a model, it is desirable to also evaluate it against other volunteer datasets to obtain a more robust representation of the population.

**FIGURE 7:**
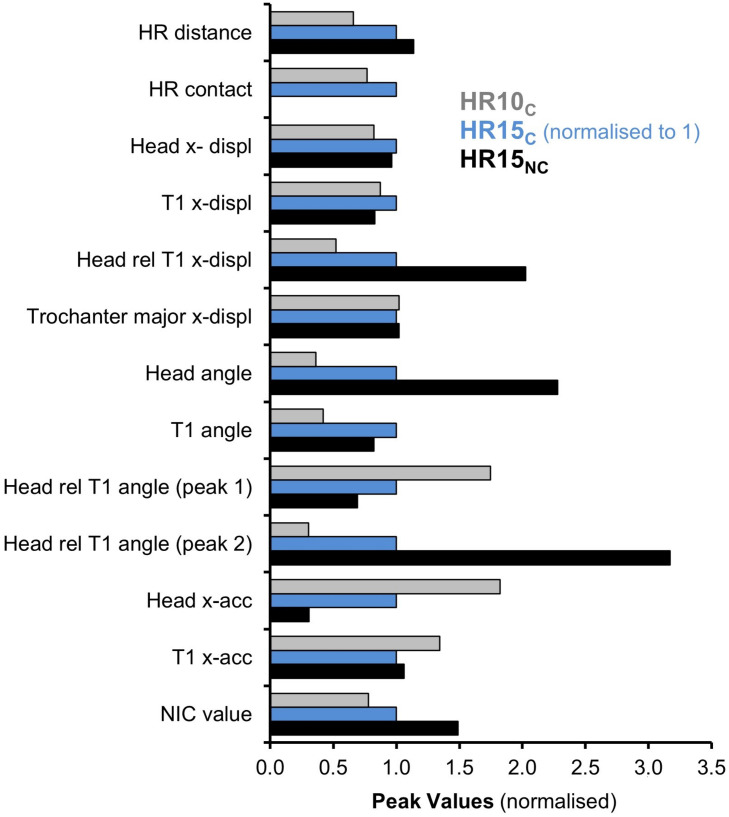
The relative HR distance and contact time; peak x-displacements (head, T1, head relative to T1, trochanter major); angular displacements (head, T1, head relative to T1); x-accelerations (head, T1) and NIC value for HR10_*C*_, HR15_*C*_ (normalised to 1) and HR15_*N*__*C*_.

The relative peak values from accelerometer signals and data from video analysis for the two configurations, HR10 and HR15, are summarised in Figure 7. The HR15_*C*_ test was used as a reference, normalised to 1 (represented by blue bars). The greatest differences between the three categories, HR10_*C*_, HR15_*C*_ and HR15_*N*__*C*_, were found for the head and head relative to T1 angular displacements. There is also a considerable difference in the T1 angular displacement for the two test configurations, HR10 and HR15, however, not between the two categories HR15_*C*_ and HR15_*N*__*C*_. Thus, the results indicate that the T1 angular displacement for the HR15 can be regarded as an upper limit for this test setup. Similar results can be seen for the head x-displacement, with a difference between the two test configurations, HR10 and HR15, this has not, however, been observed between the two categories HR15_*C*_ and HR15_*N*__*C*_. This result supports the idea that the HR15 test configuration is close to the limit of whether HR contact will occur, for this setup. The data also indicate that the initial HR distance was somewhat greater for the two volunteers in the HR15_*N*__*C*_ category, which might explain why their heads did not reach the HR. A significant increase was observed in the head relative to T1 x-displacements for increasing HR distance, HR10_*C*_, HR15_*C*_ and HR15_*N*__*C*_. In contrast, the x-displacement of the trochanter major (pelvic region) seems unaffected by the different HR configurations. The head x-acceleration decrease for increasing HR distances, HR10_*C*_, HR15_*C*_, and HR15_*N*__*C*_, may (partly) be explained by increasing head angular displacements. Furthermore, an increase of the NIC-values for increasing HR distances, HR10_*C*_, HR15_*C*_, and HR15_*N*__*C*_, was also recorded.

This study has several limitations. Due to financial constraints, the test series was limited to eight volunteers in two HR configurations. Although additional volunteers would have been valuable, this sample size is in line with other similar studies. The volunteers were young (22–29 years); an older sample might have had a somewhat different response. However, the age of the volunteers in the present study corresponds quite well to the age group with the highest whiplash injury risk (Jakobsson et al., 2000). Moreover, the outcome might have been affected by the volunteers not being exposed to the two HR configurations in a randomised order. It was decided to make the tests non-randomised to give the volunteers the option of discontinuing their participation once they had been exposed to the shorter head-to-HR distance. Furthermore, the outcome might also have been affected by the volunteers being aware of the impending impact. An “unexpected” impact was not possible to achieve, since the noise and vibrations caused by the bullet sled could be sensed. In addition, electromyographic (EMG) activity was not measured in this study. This type of measurement would potentially have given information about to what extent the volunteers were relaxed or tense at the time of impact (*T* = 0).

Philippens et al. (2002) compared the dynamic response of the 50th percentile male BioRID to volunteer and post-mortem human subject (PMHS) data and observed similar responses in low-speed rear impact tests. The dynamic response of the BioRID dummy was validated with regard to male volunteer tests in Davidsson et al. (1999), the same tests that the female volunteers in Carlsson et al. (2011) were compared to. However, the results from the latter study show that the female volunteers had a somewhat different dynamic response than the male volunteers. Similar findings have been reported in other studies (Siegmund et al., 1997; Mordaka and Gentle, 2003; Viano, 2003; Ono et al., 2006; Linder et al., 2008; Schick et al., 2008; Carlsson et al., 2012). There does not seem to be a simple way to “reinterpret” or “scale” data obtained with the BioRID II to address the female dynamic response (Carlsson, 2012). Thus, it is important that future whiplash protection systems are developed and evaluated with consideration of the female properties as well. With this study we have been able to supply new data that can be used for validation of a 50th percentile low speed rear impact female crash test dummy and/or computational models.

## Data Availability Statement

The raw data supporting the conclusions of this article will be made available by the authors, without undue reservation.

## Ethics Statement

The studies involving human participants were reviewed and approved by the Ludwig-Maximilian University in Munich, Germany Approval Reference Number 319–07 Address: Ethikkommission der Medizinischen Fakultät der LMU, Pettenkoferstr. 8a, 80336 Munich, Germany. The patients/participants provided their written informed consent to participate in this study.

## Author Contributions

AC: preparation, execution, documentation, and analysis of the test series and main author. SH: preparation and execution of the test series, internal review of the manuscript. JD: preparation of the test series, advice based on earlier experience in experimental whiplash injury research including volunteer testing and crash test dummy development, and internal review of the manuscript. SS: medical responsibility, preparation of the test series, advice based on earlier experience in experimental whiplash injury research including volunteer testing, and internal review of the manuscript. AL: EU project coordinator, contributed to the planning of the test series, advice based on earlier experience in experimental whiplash injury research including volunteer testing, and internal review of the manuscript. WH: WP-leader in the ADSEAT project, contributed to the planning of the test series, and internal review of the manuscript. MS: principal investigator, WP-leader in the two involved EU-projects, initiated the work in the present study, contributed with advice based on earlier experience in experimental whiplash injury research including volunteer testing and crash test dummy development, and contributed to the writing and internal review of the manuscript. All authors contributed to the article and approved the submitted version.

## Conflict of Interest

The authors declare that the research was conducted in the absence of any commercial or financial relationships that could be construed as a potential conflict of interest.
