# Supplementary material for: Dynamic Responses of Female Volunteers in Rear Impact Sled Tests at Two Head Restraint Distances
Source: Front Bioeng Biotechnol. 2021 Jun 8;9:684003. doi: 10.3389/fbioe.2021.684003 (PMC8217471; doi:10.3389/fbioe.2021.684003)
Supplement: Supplementary file 1 [file Data_Sheet_1.pdf]

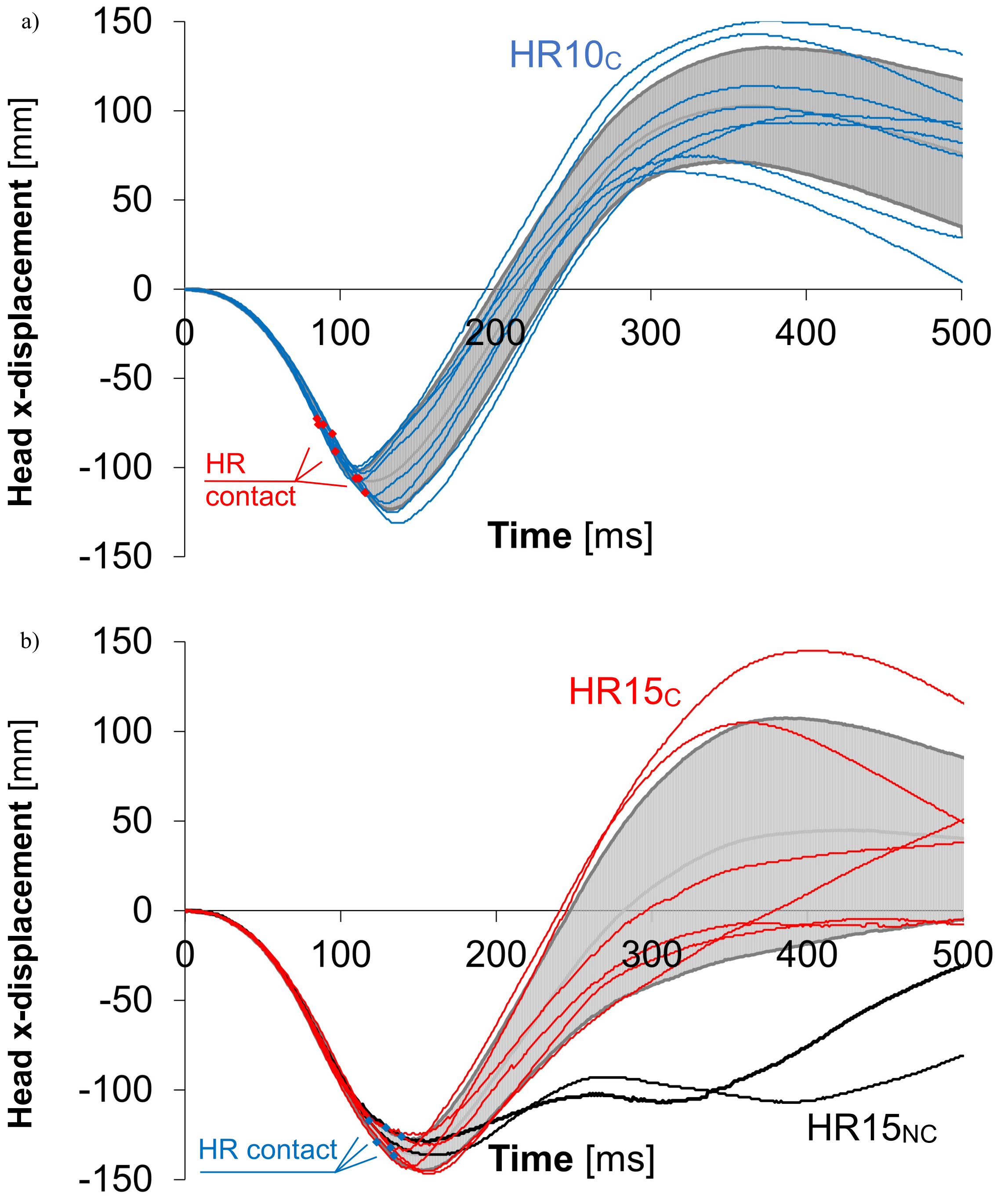

***Figure A1.1**. X-displacements of the head for near 50th percentile female volunteers. a) The eight tests in HR10$_C$ (solid blue lines) are represented by the dark grey corridor, and b) the six tests in HR15$_C$ (solid red lines) by the light grey corridor, and the two tests in HR15$_{NC}$ by the two solid black lines. The response corridors were calculated ±1SD from the average response. The head-to-HR contact times are indicated by red (HR10$_C$) and blue (HR15$_C$) dots.*

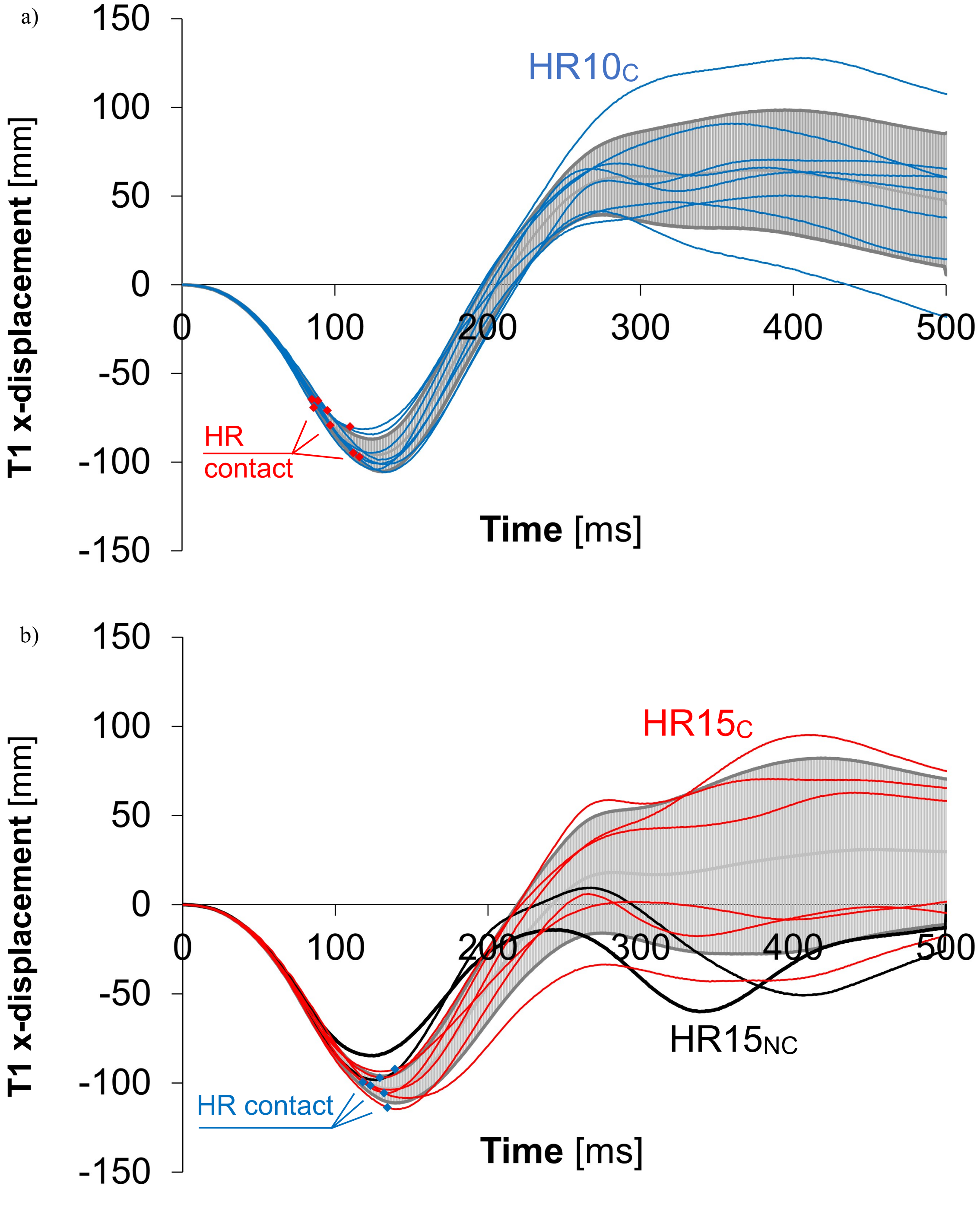

***Figure A1.2**. X-displacements of the T1 for near 50th percentile female volunteers. a) The eight tests in $HR10_C$ (solid blue lines) are represented by the dark grey corridor, and b) the six tests in $HR15_C$ (solid red lines) by the light grey corridor, and the two tests in $HR15_{NC}$ by the two solid black lines. The response corridors were calculated ±1SD from the average response. The head-to-HR contact times are indicated by red ($HR10_C$) and blue ($HR15_C$) dots.*

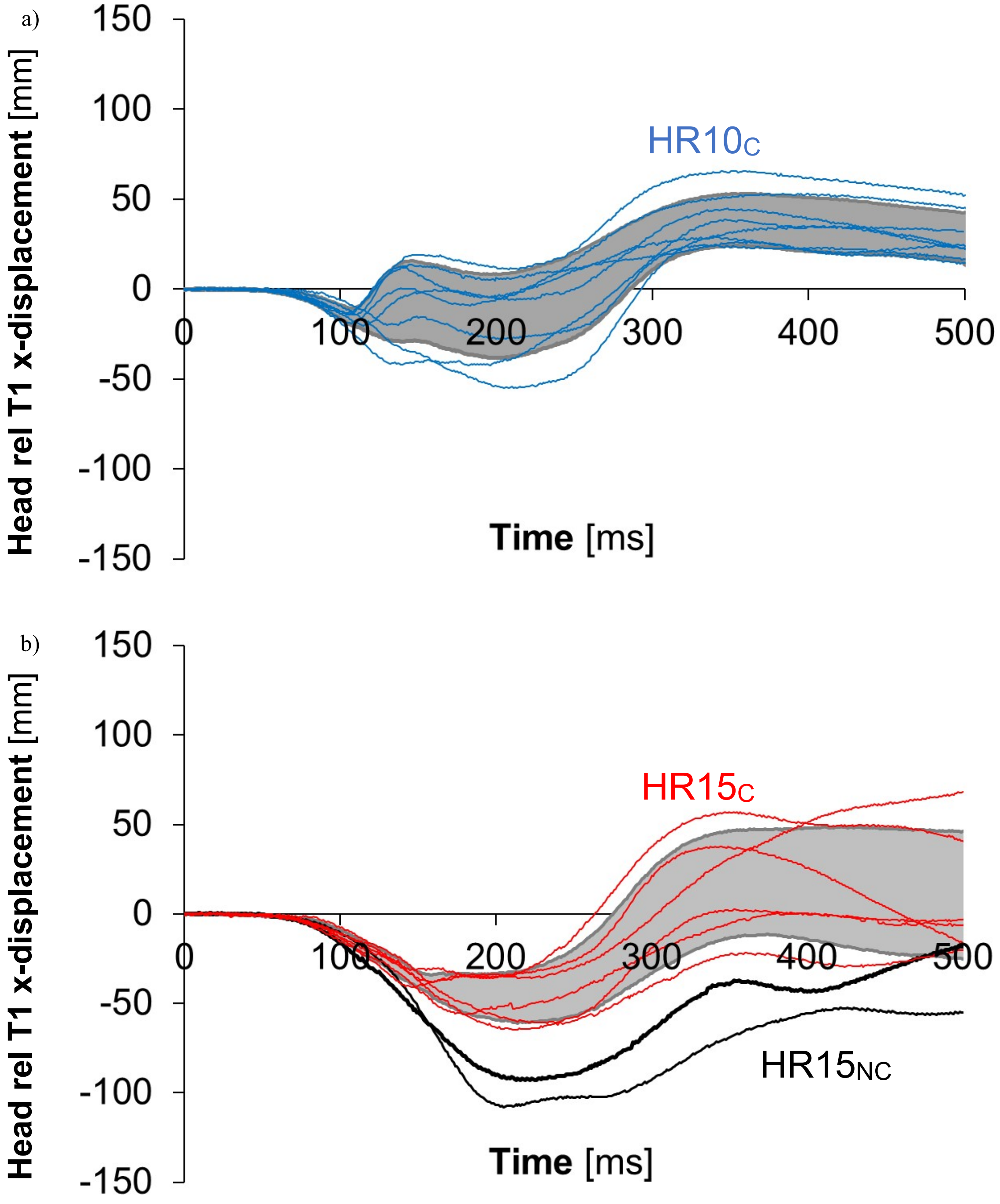

Figure A1.3. *X-displacements of the head relative to T1 for near 50th percentile female volunteers. a) The eight tests in $HR10_C$ (solid blue lines) are represented by the dark grey corridor, and b) the six tests in $HR15_C$ (solid red lines) by the light grey corridor, and the two tests in $HR15_{NC}$ by the two solid black lines. The response corridors were calculated ±1SD from the average response.*

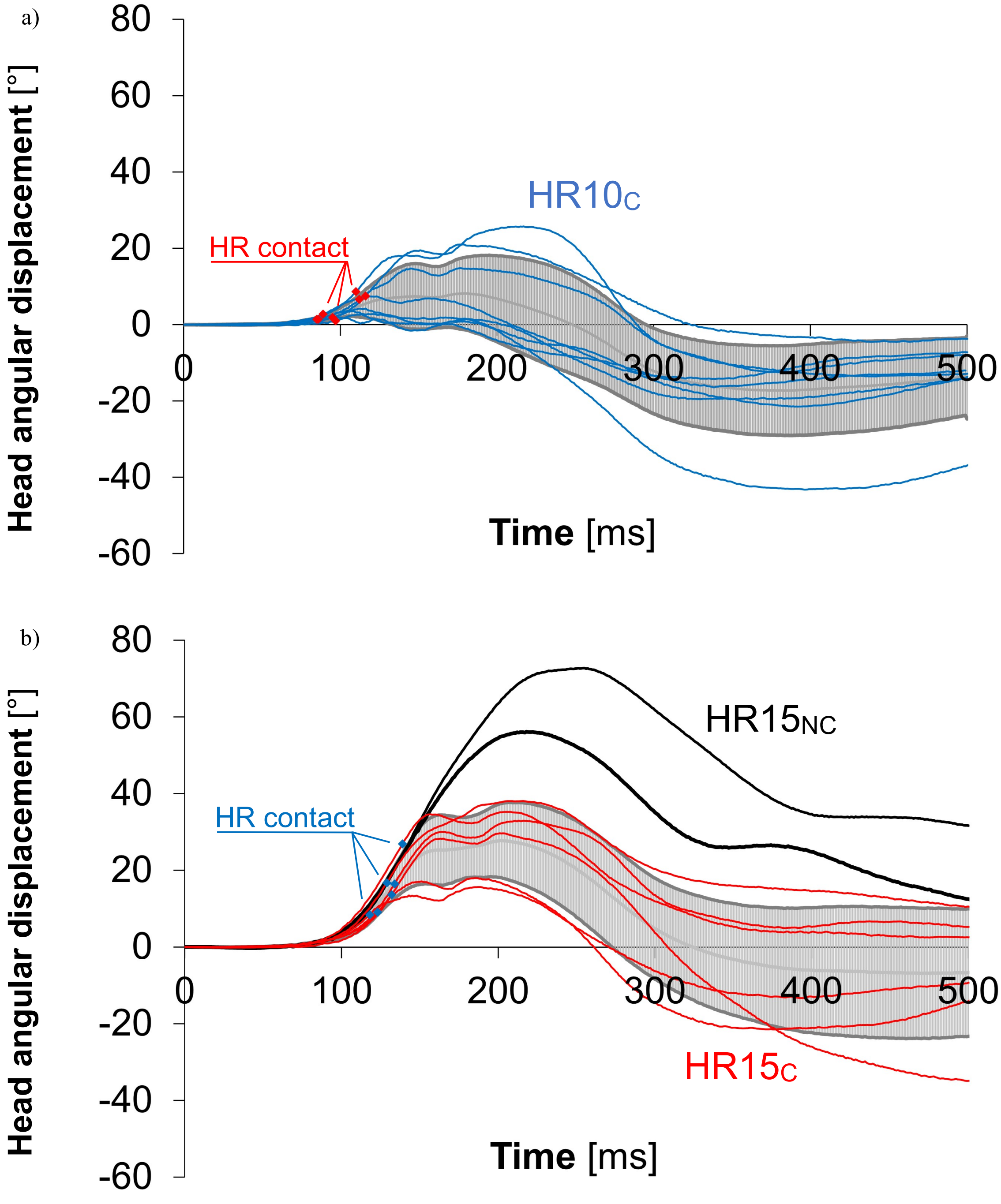

Figure A1.4. *Angular displacements of the head for near 50th percentile female volunteers. a) The eight tests in $HR10_C$ (solid blue lines) are represented by the dark grey corridor, and b) the six tests in $HR15_C$ (solid red lines) by the light grey corridor, and the two tests in $HR15_{NC}$ by the two solid black lines. The response corridors were calculated ±1SD from the average response. The head-to-HR contact times are indicated by red ($HR10_C$) and blue ($HR15_C$) dots.*

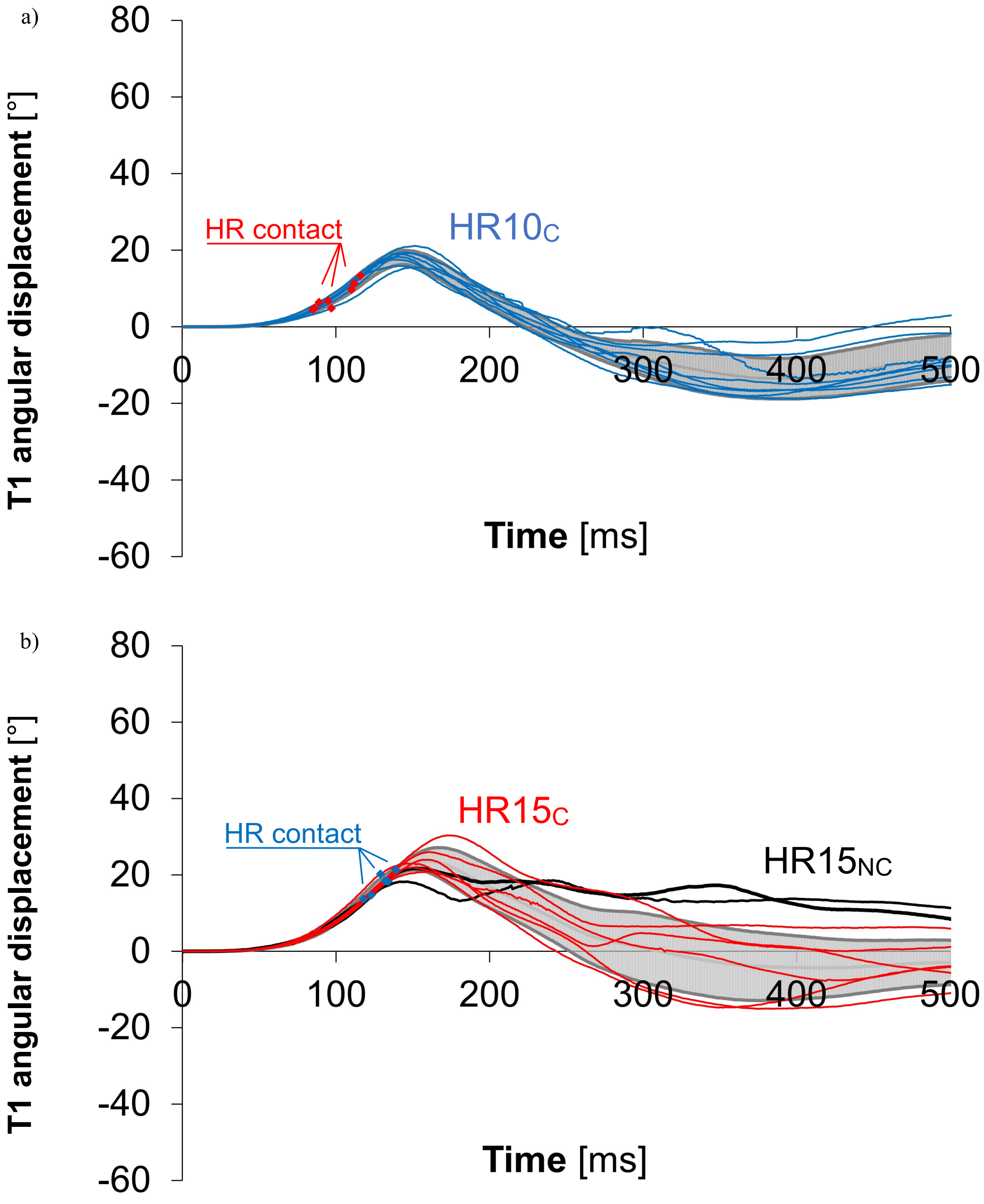

Figure A1.5. *Angular displacements of the T1 for near 50th percentile female volunteers. a) The eight tests in HR10$_C$ (solid blue lines) are represented by the dark grey corridor, and b) the six tests in HR15$_C$ (solid red lines) by the light grey corridor, and the two tests in HR15$_{NC}$ by the two solid black lines. The response corridors were calculated ±1SD from the average response. The head-to-HR contact times are indicated by red (HR10$_C$) and blue (HR15$_C$) dots.*

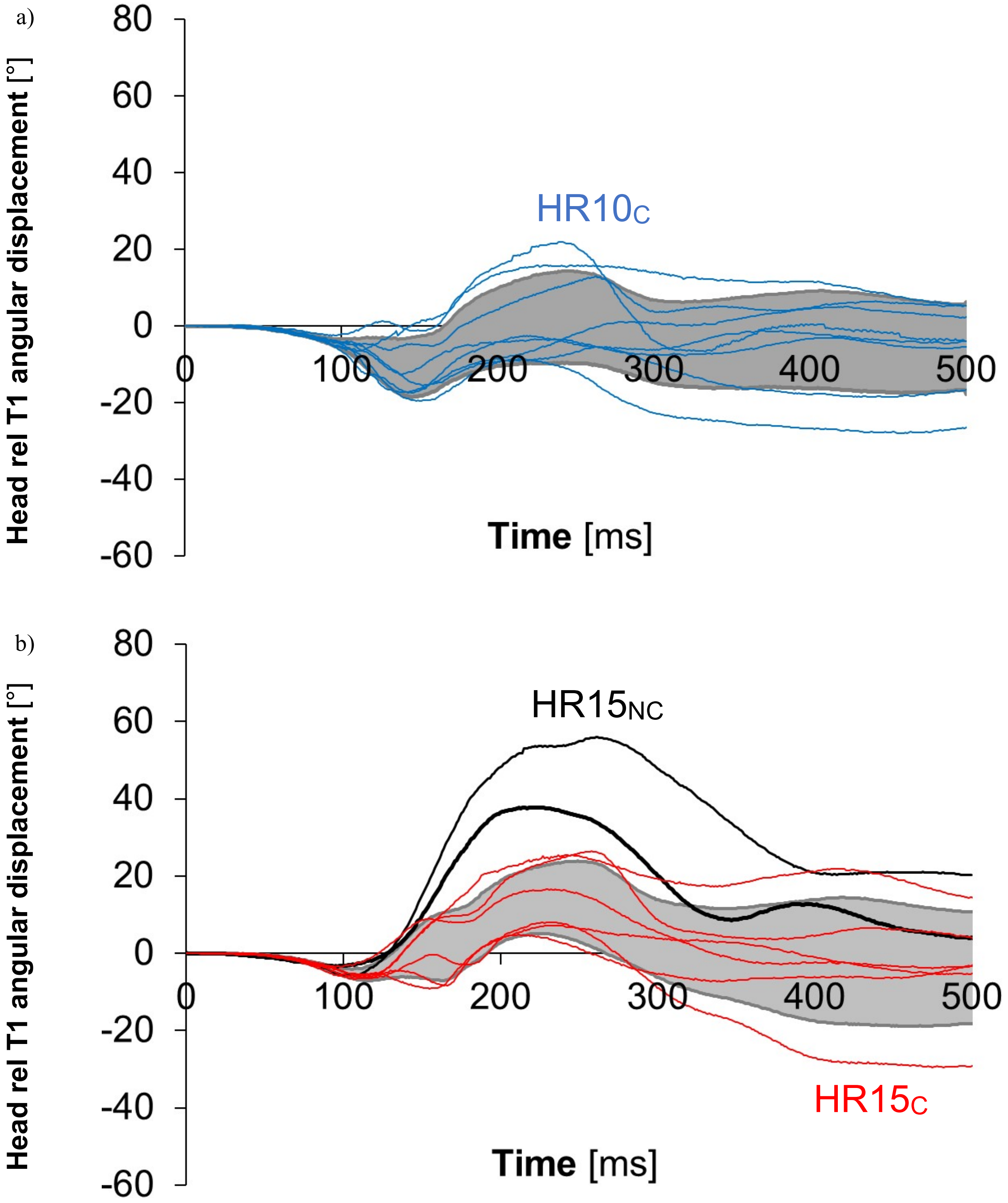

***Figure A1.6**. Angular displacements of the head relative to T1 for near 50th percentile female volunteers. a) The eight tests in $HR10_C$ (solid blue lines) are represented by the dark grey corridor, and b) the six tests in $HR15_C$ (solid red lines) by the light grey corridor, and the two tests in $HR15_{NC}$ by the two solid black lines. The response corridors were calculated ±1SD from the average response.*

a)

b)

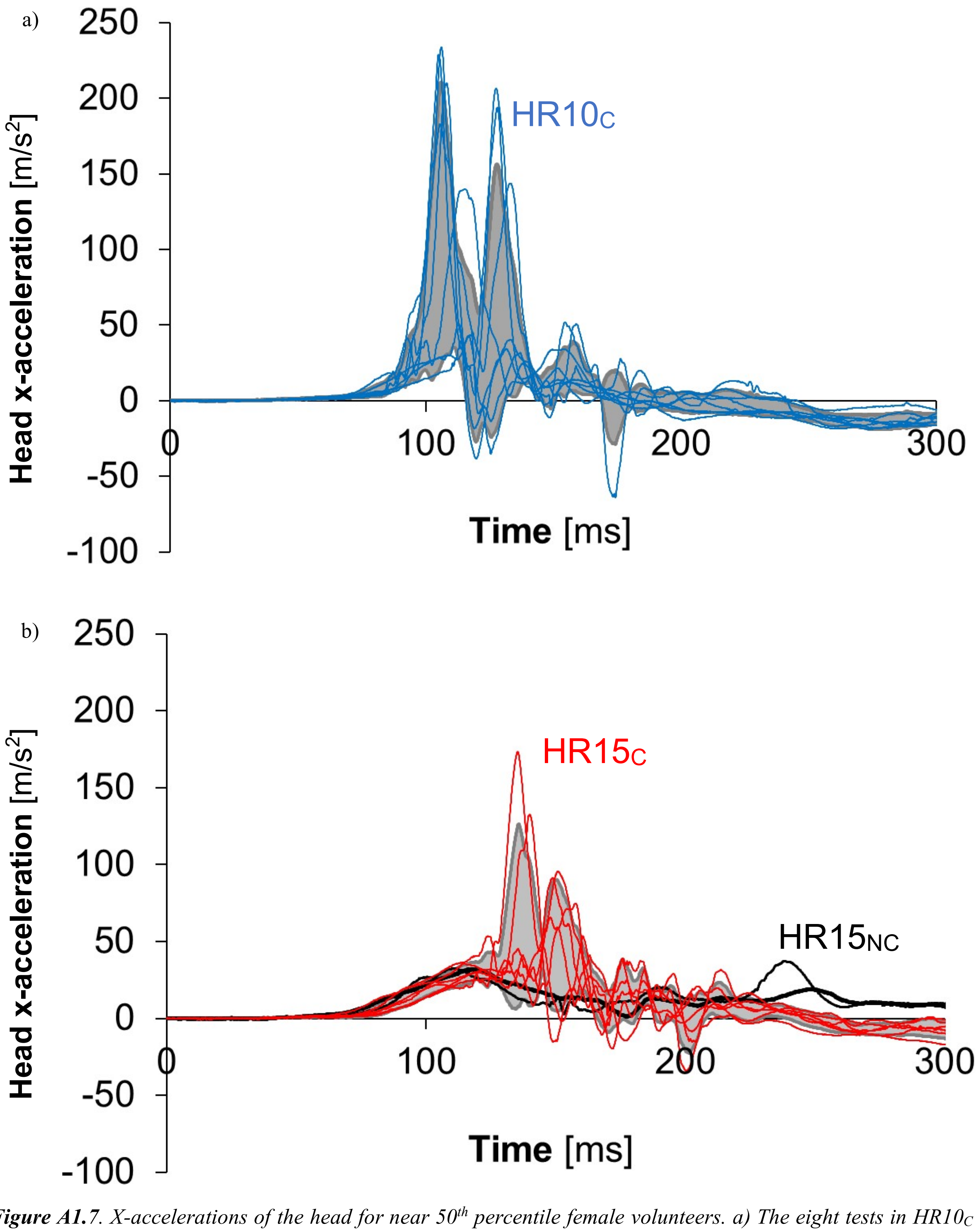

Figure A1.7. *X-accelerations of the head for near 50th percentile female volunteers. a) The eight tests in HR10$_C$ (solid blue lines) are represented by the dark grey corridor, and b) the six tests in HR15$_C$ (solid red lines) by the light grey corridor, and the two tests in HR15$_{NC}$ by the two solid black lines. The response corridors were calculated ±1SD from the average response.*

a)

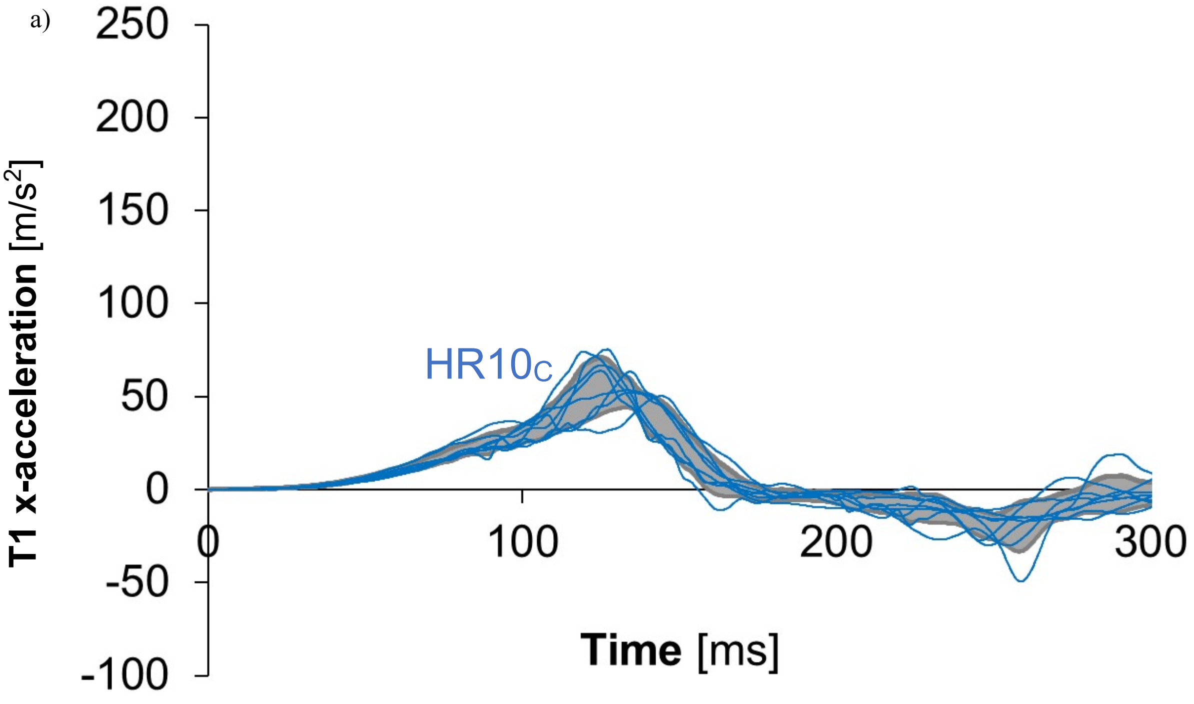

b)

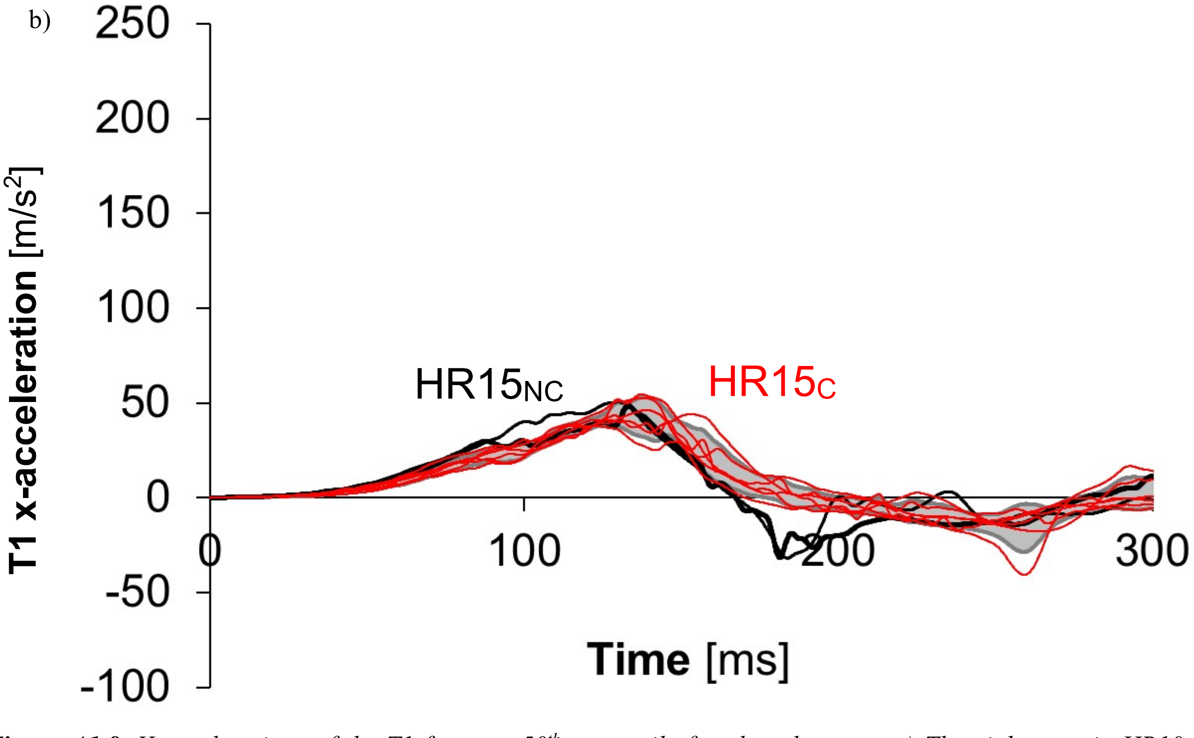

Figure A1.8. *X-accelerations of the T1 for near 50th percentile female volunteers. a) The eight tests in $HR10_C$ (solid blue lines) are represented by the dark grey corridor, and b) the six tests in $HR15_C$ (solid red lines) by the light grey corridor, and the two tests in $HR15_{NC}$ by the two solid black lines. The response corridors were calculated ±1SD from the average response.*

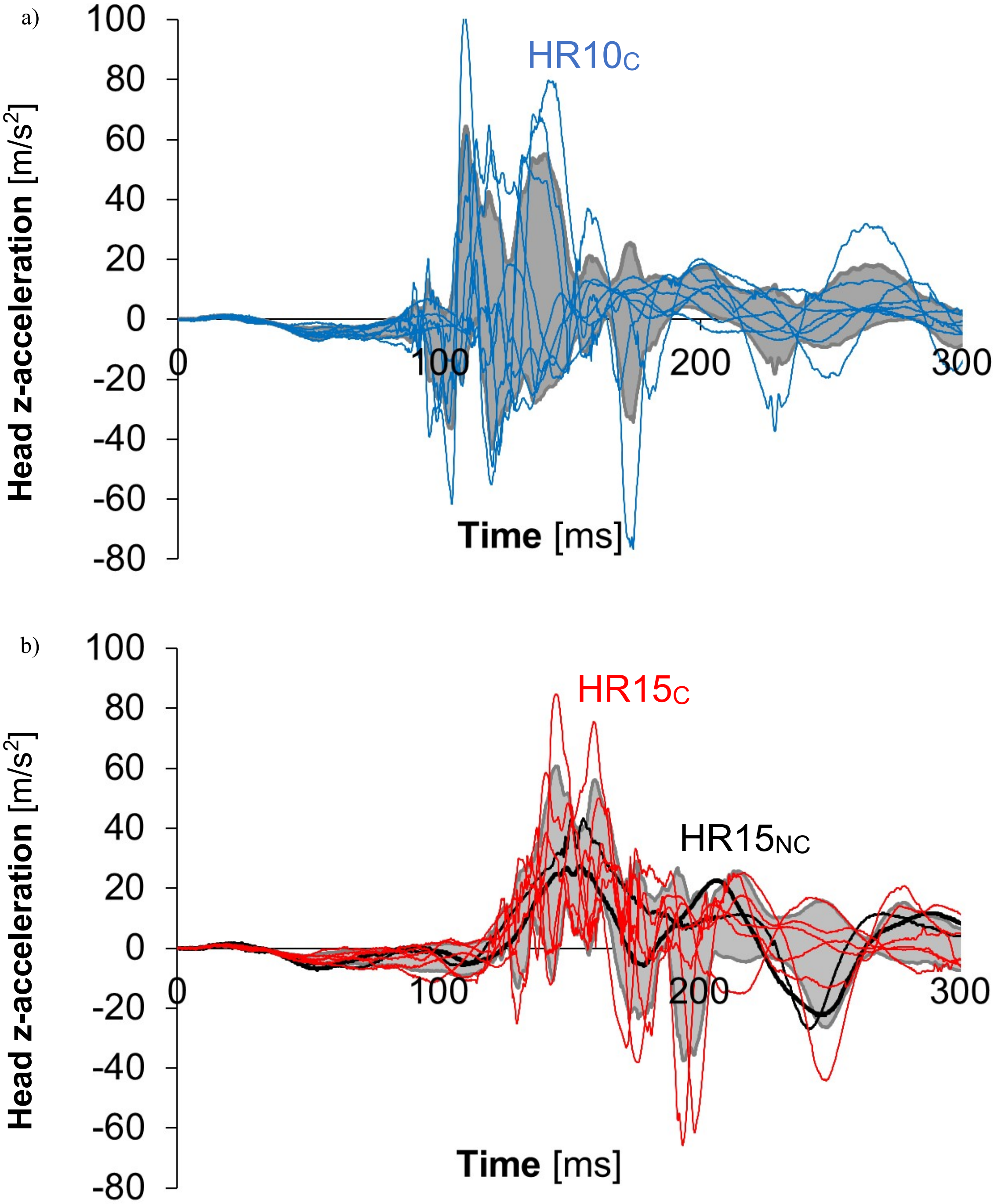

Figure A1.9. *Z-accelerations of the head for near 50th percentile female volunteers. a) The eight tests in HR10$_{C}$ (solid blue lines) are represented by the dark grey corridor, and b) the six tests in HR15$_{C}$ (solid red lines) by the light grey corridor, and the two tests in HR15$_{NC}$ by the two solid black lines. The response corridors were calculated ±1SD from the average response.*

a)

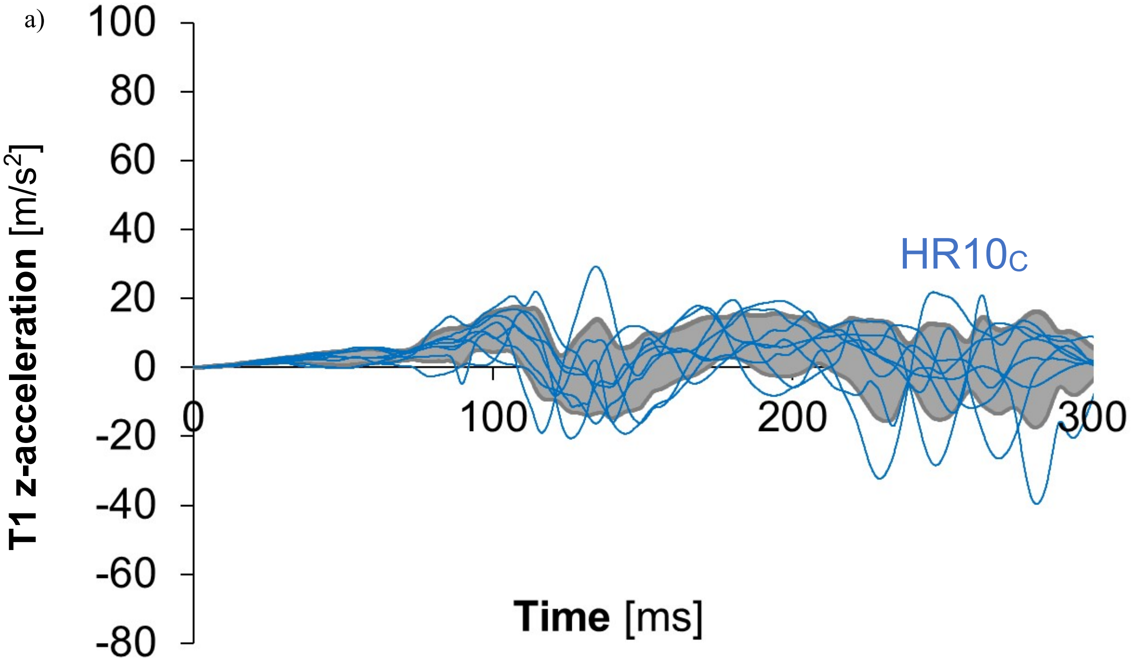

b)

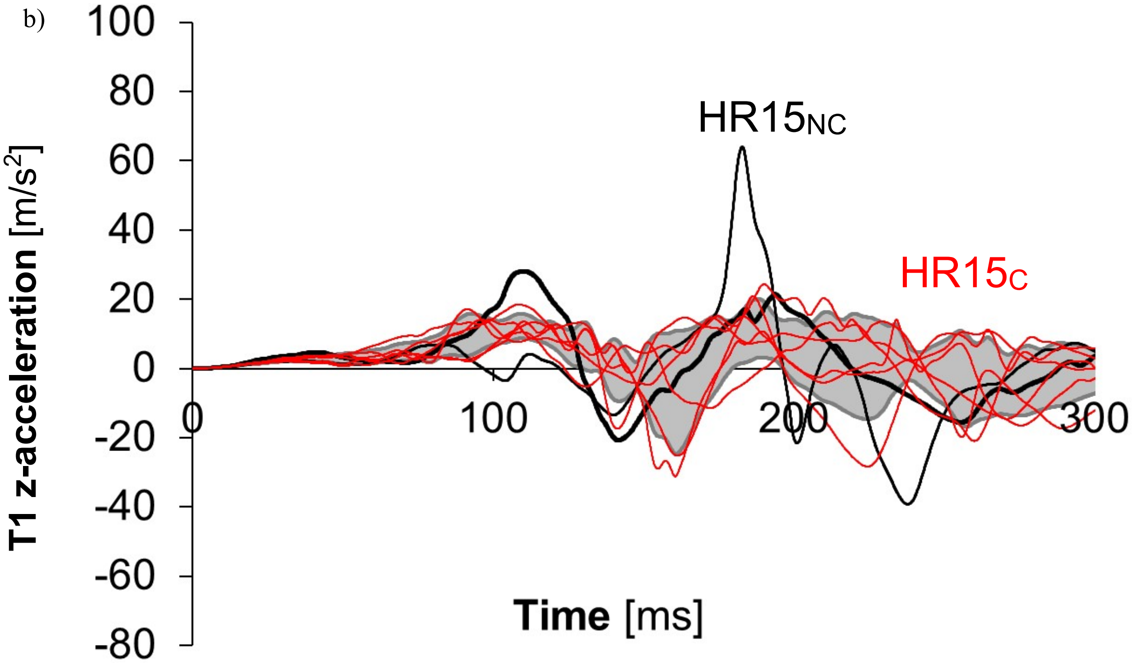

Figure A1.10. *Z-accelerations of the T1 for near 50th percentile female volunteers. a) The eight tests in HR10$_C$ (solid blue lines) are represented by the dark grey corridor, and b) the six tests in HR15$_C$ (solid red lines) by the light grey corridor, and the two tests in HR15$_{NC}$ by the two solid black lines. The response corridors were calculated ±1SD from the average response.*
